# Risk factors for non-atopic asthma/wheeze in children and adolescents: a systematic review

**DOI:** 10.1186/1742-7622-11-5

**Published:** 2014-06-06

**Authors:** Agostino Strina, Mauricio L Barreto, Philip J Cooper, Laura C Rodrigues

**Affiliations:** 1 Universidade Federal da Bahia, Instituto de Saude Coletiva, Rua Basilio da Gama s/n, 40110-040 Salvador, Bahia, Brazil; 2 St George’s University of London, Division of Clinical Sciences, Cranmer Terrace, London SW17 ORE, UK; 3 London School of Hygiene & Tropical Medicine, Faculty of Epidemiology and Population Health, Keppel Street, London WC1E 7HT, UK

**Keywords:** Non-atopic asthma, Non-atopic wheeze, Risk factors, Mould, Respiratory infections

## Abstract

**Background:**

The study of non-atopic asthma/wheeze in children separately from atopic asthma is relatively recent. Studies have focused on single risk factors and had inconsistent findings.

**Objective:**

To review evidence on factors associated with non-atopic asthma/wheeze in children and adolescents.

**Methods:**

A review of studies of risk factors for non-atopic asthma/wheeze which had a non-asthmatic comparison group, and assessed atopy by skin-prick test or allergen-specific IgE.

**Results:**

Studies of non-atopic asthma/wheeze used a wide diversity of definitions of asthma/wheeze, comparison groups and methods to assess atopy. Among 30 risk factors evaluated in the 43 studies only 3 (family history of asthma/rhinitis/eczema, dampness/mold in the household, and lower respiratory tract infections in childhood) showed consistent associations with non-atopic asthma/wheeze. No or limited period of breastfeeding was less consistently associated with non-atopic asthma/wheeze. The few studies examining the effects of overweight/obesity and psychological/social factors showed consistent associations. We used a novel graphical presentation of different risk factors for non-atopic asthma/wheeze, allowing a more complete perception of the complex pattern of effects.

**Conclusions:**

More research using standardized methodology is needed on the causes of non-atopic asthma.

## Introduction

Asthma is the most common chronic noninfectious disease of childhood and is estimated to affect 300 million people worldwide, causing a quarter of a million deaths and 15 million disability-adjusted life years (DALYs) lost annually [1]. The prevalence of asthma may have reached a plateau in many industrialized countries, but appears to be increasing in many low- and middle-income countries [2]. Asthma is a complex group of conditions, and efforts have been made to define phenotypes/endotypes based on age of onset, duration, severity, presence of allergy and other factors [3-8]. There is no agreed definition of asthma for research purposes and different research projects use different definitions, based on clinical symptoms, questionnaire data and pulmonary function tests. Asthma has traditionally been considered an allergic disease, but although allergic children are more likely to have asthma, a large proportion of children with asthma do not appear to be allergic. The proportion of asthma that can be attributable to atopy varies geographically [9,10], and clinical features are similar between atopic and non-atopic asthma [11]. Causation of asthma remains poorly understood with little consensus about the relative importance of different putative causal factors. Most studies have ignored the distinction between atopic and non-atopic disease even though these phenotypes are likely to have distinct causal mechanisms.

Recently, studies have started to investigate separately risk factors for non-atopic asthma and atopic asthma, and in so doing have raised at least two additional methodological challenges: the definition of atopy and the use of an appropriate comparison group. With respect to atopy, there is a high level of concordance between the results of skin prick test (SPT) reactivity and specific IgE (sIgE) to aeroallergens in studies done in Europe and the USA – many studies have used SPT for reasons of cost and convenience. However, in low- and middle-income countries there is often a marked dissociation between the results of SPT and allergen-specific IgE, particularly in underprivileged populations. Such an effect may relate to the immune modulatory effects of chronic infections [12,13] that target allergic effector responses rather than allergic sensitization. There is the risk, therefore, that the use of SPT to measure atopy in such populations may misclassify atopic individuals (i.e. sIgE+) as non-atopic.

The other important methodological challenge is the choice of the appropriate comparison group. Studies of risk factors for non-atopic asthma have used different comparison groups: some used all non-asthmatic children, some atopic asthmatics, and others non-atopic non-asthmatics. There are substantive consequences to this choice; this has been discussed elsewhere [14,15].

This is the first systematic review of the evidence for association between a wide range of risk factors and non-atopic asthma or wheeze in children and adolescents. Given the variations in definitions of atopy and in the comparison groups used, we present the evidence for consistent associations but do not provide a statistical summary of these associations.

## Methods

Following the reporting guidelines recommended in PRISMA [16] (Additional file 1), we conducted a systematic review of the scientific literature to identify studies that have investigated factors associated with asthma or wheeze in children and adolescents, including studies that investigated non-atopic asthma exclusively, and those presenting results separately for non-atopic asthma.

Search: The electronic database Medline was searched by AS. The search covered the period 1972 to February 25th^th^, 2014. The search algorithm applied in free text was: ((intrinsic asthma) OR (neutrophilic asthma) OR (nonatopic asthma) OR (non-atopic asthma) OR ("non atopic" asthma) OR (nonallergic asthma) OR (non-allergic asthma) OR ("non allergic" asthma) OR (nonatopic wheez*) OR (non-atopic wheez*) OR ("non atopic" wheez*)) AND (risk OR ratio). Reference lists of past reviews or meta-analyses and of publications retained for review were evaluated also. Articles in English, French, Italian, Portuguese and Spanish were read in the original language. We were inclusive in accepting studies with a wide range of definitions of asthma/wheeze based on clinical symptoms or questionnaire.

Eligibility criteria: Titles and abstract of the articles identified with the initial search were screened, and articles were excluded from further analysis based on title (not relevant) or abstract (unequivocal presence of excluding features). Excluding features were: article languages other than those specified, adult asthma/wheeze, occupational asthma/wheeze, study designs other than cohort, case–control or cross-sectional designs, genetic studies, definition of atopy other than measurement of sIgE, skin-prick test (SPT) or both, and studies without a non-asthmatic comparison group (i.e., those that did not compare asthmatics with non-asthmatics or explored risk factors for exacerbations, severity or mortality in asthmatic populations). Review and meta-analysis articles were used as sources of references only. No contact was made with study investigators.

**Figure 1:**
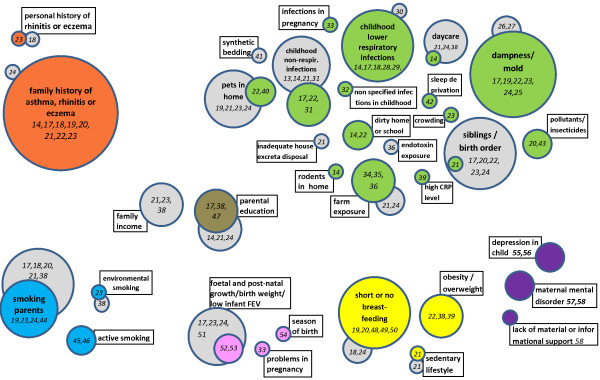
**Evidence base for increase in non-atopic asthma in the following domains: infections/humidity/dirt (green), history (orange), nutrition (yellow), psychosocial (violet), smoking (blue), pregnancy and perinatal issues (pink), and socio-economic status (brown); evidence not found (grey).** Risk factors are displayed as circles of sizes proportional to the number of studies. Digits inside the circles are the relevant reference numbers.

Articles not excluded in the first screen were read by one author (AS), and either were excluded based on the criteria listed above, or had information saved in a working table including: author(s) name, year of publication, geographic region, size and age of the study population, study design, way of ascertainment and definition of the endpoint (asthma/wheeze), definition of atopy, definition of comparison group, adjustment for confounding, and effect measures (see Additional file 2). Any doubts and uncertainties where discussed with another author (LR). Results of studies in which only univariate analysis was performed were included. We considered that no statistically significant association with outcome was found for those factors for which data were presented in descriptive or univariate tables but were not retained in multivariate analysis.Risk factors were grouped in domains: family history, smoking, socioeconomic factors, pregnancy and perinatal factors, nutritional factors, psychosocial factors and dirt/infection factors. Because many different exposures were studied, we present an overview of the results in a summary figure (Figure 1) using a novel graphic presentation (Frequency of Risk factors Circles or FRiskC). FRIskCs displays risk factors as circles of sizes proportional to the number of studies. For each factor, a colour circle represents the number of studies that found a statistically significant association and the grey circle the number of studies that did not. Domains were colour-coded. Digits inside the circles are the relevant reference numbers.

Summary measures were not estimated for each factor because of the diversity of methods used between studies.

## Results

**Figure 2:**
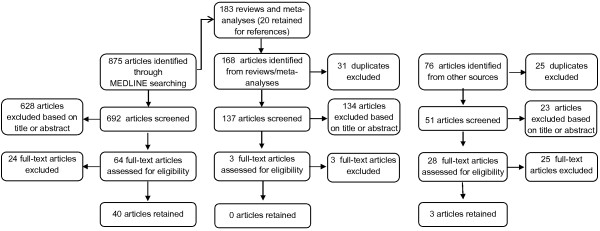
Flowchart of the search.

The initial search of the literature identified 875 articles, to which 244 more were added from reference lists of reviews/meta-analyses and articles retained for review. After eliminating duplicates and performing the first screening, based on title or abstract, 95 articles were retained for full-text reading, after which 52 were excluded for different reasons and 43 articles were retained for review (Figure 2).

Figure 1 displays risk factors by domain and whether the studies showed a statistically significant association; ORs and 95% CI are shown in Table 1 and Additional file 2. Of the 30 individual or grouped factors, only three were investigated in a large number of studies and showed very consistent associations with non-atopic asthma/wheeze: family history of asthma, living in a home with damp or humidity, and history of respiratory infections in childhood. Another two factors that were investigated in numerous papers, were consistently not associated with non-atopic asthma/wheeze: family income and birth order. For all other factors, either the studies were less numerous or showed conflicting results.

**Table 1:** Factors associated with non-atopic asthma/wheeze by domain, OR and 95% CI

***Reference***	**Domain**		**OR**	**(95% CI)**
	**Grouped risk factors**	**Individual risk factors**		
	**History of asthma, rhinitis or eczema**			
	Family history			
[14]		Family history of asthma, rhinitis or eczema	1.91	(1.25–2.92)
[17]		Family history of asthma, rhinitis or eczema	5.4	(2.5–11.7)
[18]		Family history of asthma, rhinitis or eczema	4.08	(1.85–9.00)
[19]		Family history of asthma, rhinitis or eczema	3.63	(2.33–5.66)
[20]		Family history of asthma, rhinitis or eczema	3.45	(1.18–10.10)
[21]		Family history of asthma, rhinitis or eczema	3.24	(2.42–4.32)
[22]		Family history of asthma, rhinitis or eczema	2.35	(1.23–4.52)
[23]		Family history of asthma, rhinitis or eczema	1.74	(1.30–2.32)
	Personal history			
[23]		Personal history of rhinitis	2.49	(2.10–2.95)
[23]		Personal history of eczema	2.16	(1.63–2.88)
	**Infection/humidity/dirt**			
	Dampness/mold			
[17]		Current damp in the household	2.7	(1.4–5.2)
[19]		Current or past damp	1.78	(1.10–2.89)
[22]		Current damp in the household	2.45	(1.11–5.40)
[23]		Current damp in the household	1.48	(1.06–2.08)
[23]		Past damp in household	2.44	(1.73–3.44)
[24]		Current damp in the household	2.70	(1.16–6.30)
[25]		Current damp in the household	1.54	(1.33–1.77)
	Childhood lower respiratory infections			
[14]		Respiratory symptoms < 8 days	1.54	(1.01–2.36)
[14]		Respiratory symptoms ≥ 8 days	4.87	(2.26–9.76)
[17]		Bronchiolitis	14.5	(7.0–30.0)
[18]		Recurrent chest infections	3.99	(1.78–8.92)
[28]		Wheezing LRI	4.10	(2.48–6.77)
[29]		Seropositivity to *Chlamidia pneumonia* (in girls)	2.39	(1.25–4.57)
	Childhood infections other than LRI			
[22]		Croup	2.80	(1.44–5.43)
[22]		Otitis	1.99	(1.02–3.88)
[31]		Anti–*A.lumbricoides* IgE	3.07	(1.13–8.35)
[17]		Ascaris infection	3.1	(1.1–6.6)
	Non–specified infections in childhood			
[32]		5+ episodes of fever	11.48	(5.95–22.12)
[32]		6+ antibiotic courses	24.29	(11.86–49.76)
	Infections in pregnancy			
[33]		‘Flu’ during pregnancy	2.28	(1.15–4.52)
[33]		Fever during pregnancy	2.18	(0.98–4.86)
	Farming exposure			
[34]		Living in a farm	0.45	(0.32–0.63)
[35]		Use of silage	0.55	(0.31–0.98)
[36]		Exposure to farming	0.43	(0.19–0.97)
[14]		Daycare	1.52	(1.01–2.29)
	Dirty home or school			
[14]		Infrequent household cleaning	2.49	(1.27–4.90)
[22]		Dirty school	2.50	(1.28–4.89)
[23]		One–room house	2.24	(1.48–3.39)
[21]		Older siblings	0.39	(0.25–0.62)
[39]		High CPR	1.45	(1.61–1.81)
	Pets in home			
[22]		Cat or dog when infant	2.17	(1.16–4.04)
[40]		Cat and dog in early childhood	3.66	(1.50–8.93)
[14]		Rodents in home	1.68	(1.21–2.34)
[42]		Sleep deprivation (and asthma at age 6 years)	1.87	(1.08–3.25)
[42]		Sleep deprivation (and asthma at age 14 years)	2.18	(1.15–4.13)
	Pollutants/insecticides			
[20]		Insecticide (dichlorodiphenyldichloroetylene)	2.49	(1.00–6.19)
[43]		NO_x_ from road traffic	2.4	(1.0–5.6)
	**Smoking**			
	Smoking parents			
[23]		Mother smoking during pregnancy	1.43	(1.08–1.89)
[24]		Mother smoking during the 1^st^ year of life	1.74	(1.17–2.58)
[19]		Mother smoking in general	1.67	(1.04–2.68)
	Active smoking			
[45]		Active smoking by the adolescent	17.1	(4.9–60.1)
[46]		Active smoking by the adolescent	3.92	(2.01–7.65)
[23]		Environmental smoking	1.63	(1.28–2.09)
	**Socioeconomic factors**			
	Parental education			
[17]		High parental education	0.3	(0.1–0.9)
[47]		High parental education	0.65	(0.43–0.99)
	**Nutrition**			
	Obesity/overweight			
[22]		per 5 units increase of BMI	1.55	(1.02–2.36)
[39]		Obesity	2.46	(1.21–5.02)
[21]		Sedentary life	1.51	(1.06–2.16)
	Breastfeeding			
[20]		Any breastfeeding	0.34	(0.17–0.69)
[48]		Any breastfeeding	0.52	(0.27–0.98)
[49]		Any breastfeeding in non–affluent countries	0.69	(0.53–0.90)
[19]		Short period of breastfeeding	1.80	(1.11–2.92)
[50]		Short period of breastfeeding	2.95	(1.31–6.66)
	**Pregnancy and perinatal issues**			
[52]*		Lower early fetal growth trajectory	0.90	(0.81–1.00)
[54]		Born in autumn	2.35	(1.14–4.83)
	Pregnancy			
[33]		Threatened abortion/premature labour	1.66	(0.96–2.86)
[33]		Exposure to isoxsuprine	1.87	(1.12–3.12)
	**Psychosocial**			
	Depression in child			
[55]		Depression in child	2.47	(1.12–5.44)
[56]		Depression in child	2.90	(1.46–5.73)
	Maternal mental disorder			
[57]		Maternal anxiety	1.78	(1.24–2.57)
[58]		Maternal mental disorder	1.73	(1.17–2.55)
	Social support			
[58]		Informational support	0.60	(0.40–0.90)
[58]		Material support	0.63	(0.42–0.95)

*RR (95% CI).

Family history of asthma, rhinitis or eczema showed a consistently high and significantly positive association with asthma or wheeze in eight studies, with magnitudes of association ranging from OR = 5.4 to OR = 1.74 [14,17-23]. One study also detected risks associated with a personal history of rhinoconjunctivitis and of eczema [23].

After family history of asthma, the second most frequently studied risk factor for which a consistent and positive association with the outcome was found was presence of damp in the household, either observed currently [17,22-24], in the past [23], or in either periods [19,25]. One study detected a non-significant risk [26], and another showed no effect [27].

Many studies investigated the effect of infections and related factors. Five studies reported a positive association with lower respiratory symptoms or infections (LRI) in early childhood, including bronchiolitis at age 2 years [17], wheezing LRI at age 1 year [28], recurrent chest infections at 2 years [18], respiratory symptoms by 4 years of age lasting less than 8 days, or 8 days or more [14], and, in girls, but not in boys, seropositivity to *Chlamidia pneumoniae* at 4 years of age [29]. One study found no association of non-atopic wheeze at 5 years of age with wheezy or febrile viral LRI in the first year of age [30].

The effect of infections in childhood other than lower respiratory infections was less consistent. A study found a positive effect of croup and of otitis [22].The presence of anti-*Ascaris lumbricoides* IgE was a risk for non-atopic asthma in one study [31], while in the same study active infections (i.e., stool positive) with *A.lumbricoides* or *Trichuris trichiura* were not, whereas Ascaris infection was a risk factor for non-atopic wheeze in another study [17]. Three studies failed to detect significant risks associated with diarrhea episodes in infancy [14], heavy parasite burdens with *T.trichiura* presently [21], or burden of past or present infections of different etiologies [13].

Unspecified infections, expressed as number of episodes of fever or number of antibiotic courses in childhood, represented large and significant risks in one study [32]. ‘Flu’ and fever during pregnancy were also positively associated with non-atopic asthma [33].

Studies of the effect of farming exposures showed contradictory results: of five studies identified, three from Central and Northern Europe reported a significant and similar protective effect [34-36]. A similar effect as in the latter study was found in a different analysis in the same population [37]. Two studies failed to find any effect of farming exposures [21,24]. The only study that considered endotoxin exposure found a non-significant risk [36].

Four studies considered the effect of daycare [14,21,24,38], but only one showed a significant associated risk [14]. Among other conditions related to or associated with infections in childhood, presence of visible dirt at home or school was reported as a risk factor in two studies with similar results [14,22], but inappropriate household excreta disposal was not [21]. Crowding was found also to be significantly associated with asthma [23], whereas, of the six studies evaluating the effect of sibship size and birth order [17,20-24], only one found a significant protective effect of having older siblings [21]. Increased levels of C-reactive protein (CRP), a marker of systemic inflammation, yielded a significant risk [39]. Other conditions potentially associated with infections are keeping household pets [19,21-24,40], the use of synthetic bedding [41], and the presence of rodents in the household [14]. Among studies that examined these conditions, only three detected significant risks, in two, associated with cats and/or dogs during early life [22,40], and in one, with rodents [14]. Frequent waking at night during the first 3 years of life, possibly related to respiratory infections, was associated with non-atopic asthma at ages 6 and 14 years [42].

Two studies examined the effects of environmental pollutants and found significant positive associations: prenatal exposure to the insecticide dichlorodiphenyldichloroetylene [20], and exposure in first year of life to NO_x_ from road traffic [43].

Parental smoking was extensively studied but showed contradictory findings: statistically significant risks were detected in 4 of 9 studies [17-21,23,24,38,44]. These included maternal smoking during pregnancy [23] or during the 1^st^ year of life [24], mother smoking in general [19], and heavily smoking parents (67% among non-atopic asthmatics vs 40% among non-asthmatics) [44]. Active smoking by the adolescent was identified as a significant risk in two studies [45,46]. Inconsistent results were reported for second-hand smoking: one study estimated a risk [23], whereas another one reported no effect [38].

Among factors representing socioeconomic circumstances of the child’s family, parental education was evaluated in six studies showing conflicting effects: three studies found no association [14,21,24], two found a protective effect [17,47], and one found a risk (parental education level was statistically significantly higher among non-atopic asthmatic than non-asthmatic children, 12.2 years vs 12.0 years, P < 0.05) [38]. The three studies evaluating income showed no significant effects [21,23,38].

All three studies addressing the effect of obesity/overweight observed increased risks: with 5 units increase of BMI [22], obesity [39], and mean BMI percentile (68.5 in non-atopic asthmatic children vs 57.7 in non-asthmatic children, P < 0.05 [38]). Sedentary lifestyle was identified as a risk factor [21].

Breastfeeding was investigated in seven studies. Three observed a significant protective effect of any breastfeeding [20,48] (one study found an association in non-affluent countries only [49]), and two others found a risk associated with short period of breastfeeding [19,50]. A further two studies did not detect a significant effect of breastfeeding [18,24]. One study did not find an association in affluent countries [49].

There was no effect of prematurity [17,23], low birth weight [24] nor birth weight ≥ 4510 g [51]. However, a lower early fetal growth trajectory (lower head circumference growth velocity between 11 and 18 weeks) [52] and a lower lung function in early infancy (mean (95% CI) FEV_0.4_ 123.7 (114.6-133.5) ml in non-atopic wheezers vs 139.0 (132.2-146.2) ml in non-atopic non-wheezers) [53] were associated with non-atopic wheeze. In a study done in Northern Europe, being born in the autumn was associated with an increased risk [54]. Threatened abortions and exposure to isoxsuprine, a tocolytic drug, were both independently associated [33].

Two studies reported associations with depression in the child and in both the risk was higher in girls, and lower and not significant in boys [55,56]. A study reported the impact of maternal anxiety [57], and another one of maternal mental disorder [58]. The same study found also a protective effect of social support, which was highest for informational support and material support.

## Discussion

In summary, 43 studies were identified that investigated 30 grouped or individual risk factors for non-atopic asthma. Consistent and strong associations with the risk of non-atopic asthma were observed only for 3 factors: family history of asthma, rhinitis or eczema [14,17-23], dampness or mold in the household [17,19,22-25], and lower respiratory infections in childhood [14,17,18,28,29]. A brief period or no breastfeeding also showed a strong, albeit less consistent, association. Fewer studies investigated the effects of overweight/obesity and of psychological or social factors, but those that did showed consistent findings.

Family history of asthma was consistently associated with non-atopic asthma, as it has been reported for atopic asthma [14,19,22,23]. This is in agreement with genetic studies that have identified SNPs predicting both atopic and non-atopic asthma, but may also indicate the effects of a shared environment by families [59].

Equally consistent is the reported association between non-atopic asthma and indoor dampness or mold in the household [17,19,22-25], and is consistent with evidence from studies and meta-analyses showing a wide variety of respiratory effects of such exposures including the development of asthma/wheeze and exacerbations in both atopic and non-atopic individuals including infants [60]. Several plausible biological mechanisms have been proposed to mediate these effects including the induction of inflammatory, cytotoxic and immunosuppressive responses to components of microbes or fungi [60].

Wheezing related to respiratory viral infections in early life has been linked to the development of asthma at school age [61-63], although it is still matter of controversy whether lower respiratory tract infections (LRI) and asthma share a common (genetic) predisposition [64], or whether LRIs have a causal role in asthma development through impaired epithelial barrier or anti-viral immune responses or other underlying mechanisms that cause damage to, and remodeling of, the airways in susceptible children [65]. It has been suggested that exposure to an ‘aggressive’ environment, with widespread viral respiratory infections and high levels of environmental pollution, such as observed among urban poor populations in Latin America, may lead to enhanced inflammatory responses and respiratory symptoms that appear to be dissociated from atopy [66].

The effects of exposures to helminth parasites on asthma/wheeze is unclear and may vary according to type of helminth, age of first infection and infection intensity [67,68]. A consistent observation is the strong inverse association between active helminth infections and prevalence of SPT [69,70]. The modulatory effects of chronic childhood infections such as those caused by helminths on SPT prevalence [13] may add to the measurement error for atopy, because definitions of atopy based on SPT in endemic populations may lead to significant misclassification of atopy, particularly if sIgE has better explanatory value for the allergic mechanisms that mediate atopic asthma.

Farming exposures were protective in some studies [34-36], although not all studies demonstrated an association [21,24].

All but one of the studies that evaluated the number of siblings failed to find an effect, despite the protective association frequently detected when asthma (irrespective of atopy) is considered [71]. The apparent inverse association with asthma overall might be explained by the inverse association between a greater number of siblings and prevalence of atopy in populations where most asthma is atopic. The presence of household pets had no effect on non-atopic asthma except in two studies [22,40].

Almost all studies evaluating breastfeeding found a consistent, protective effect on non-atopic asthma [19,20,48-50] (one study observed an effect only in univariate analysis [18]). Interestingly, all but one studies found that the effect was on non-atopic but not on atopic asthma, and one study observed an effect only on non-atopic asthma in less affluent countries [49]. The role of breastfeeding in causation of asthma remains controversial [72-74], and may differ by atopic phenotype [49,74].

Surprisingly few studies explored the effects on non-atopic asthma of obesity/overweight [75]. Studies done, principally in adults, show that risk is increased particularly among non-atopics [76], as was observed among children in two of three studies included in the present review [38,39]. It has been suggested that these effects may be mediated through factors other than the induction of eosinophilic airway inflammation [76], and that a state of systemic inflammation may be a key factor in mediating the effects of overweight/obesity [75].

The results of parental smoking were contradictory, reflecting a complex picture of the association of parental smoking with asthma [77]. Some studies reported a risk, whereas others did not, which could be a true finding, or could be explained by parents of asthmatic children stopping smoking.

Level of parental education showed mixed effects in some studies [17,38,47], and no effect in others [14,21,24]. No effect of family income was observed [21,23,38].

Few studies have examined the associations between non-atopic asthma and child depression, maternal psychological disorders or social support, but all show an apparent effect in diverse settings. Two cross-sectional studies reported an association of depression or abnormal emotional symptoms with non-atopic asthma, but not with atopic asthma [55,56]. In both studies, the effect was found in girls, but not in boys, and in one study it persisted after controlling for abdominal adiposity [55]. It has been suggested that higher levels of leptin may link non-atopic asthma with psychological disorders: levels of leptin are elevated in girls with asthma, in non-atopic-asthmatics, and also in children with symptoms of depression [55]. A study found an association between prenatal maternal anxiety and asthma in childhood, an effect that was stronger in non-atopics than atopics and that was greater the more severe the symptoms [57]. Maternal mental disorders, including depression and anxiety, were associated, independent of maternal history of asthma and the child’s age, with both non-atopic and atopic asthma [58] . An independent protective effect of social support was found in the same study, but only for non-atopic wheezing.

This review exposed the wide heterogeneity between studies with respect to methodology: studies used different comparison groups (non-atopic non-asthmatic children, all non-asthmatic children, atopic asthmatic children), different definitions of atopy (skin test positivity to allergens, presence or levels of allergen specific IgE using different cut-off points for positivity and different panels of allergens), different definitions of asthma/wheeze (doctor’s diagnosis of asthma used in some studies may select more severe cases, whereas use of asthma-related symptoms such as wheeze adopted in other studies may lead to inclusion of milder cases), and different study designs with different ability to establish causality. Some studies were underpowered.

Despite the number of studies found, their heterogeneity with respect to the above did not allow separate analyses by specific characteristics (e.g. comparison group used), nor to explore how much of the variation is due to the methodological diversity. And yet, a few, remarkably consistent risk factors (respiratory infections in childhood, family history of asthma, eczema or rhinitis, and presence of mold or damp in the household) were identified.

Strengths and Limitations. We developed a novel graphic presentation of data, which we believe enhances the perception of the complex pattern of effects. Given the highly heterogeneous methodologies used in the studies, a meta-analysis was not performed even for the risk factors investigated in a sufficient number of studies. Maybe the most important finding of this review is the very marked heterogeneity of methods used in the existing studies, which in our view has delayed scientific progress in this field.

## Conclusion

Research in asthma and the ability to compare between studies clearly requires a consistent and standardized methodological approach. Allergen-specific IgE levels seem to provide a more reliable and stable characterization of atopy than SPT positivity, particularly in populations living in low- and middle-income countries where the two measures are dissociated [12,13], and should, therefore, be preferably used to define atopic status in studies of asthma epidemiology. The debate is ongoing on which is the best comparison group (and on the implications for the interpretation of findings) [14,15].

### Recommendations

Research using agreed, rigorous methodology is needed to elucidate the pattern of association and causal mechanisms underpinning non-atopic asthma.

## Competing interests

The authors declare that they have no competing interests.

## Authors’ contributions

AS and LCR designed the study, AS conducted the search and read the abstracts and the papers, AS and LCR classified the papers, AS conducted the analysis, AS, MLB, PJC and LCR interpreted the results, AS wrote up the manuscript, and MLB, PJC and LCR contributed to writing. All authors read and approved the final manuscript.

## Supplementary Material

Additional file 1 PRISMA 2009 checklist. Click here for file

Additional file 2 Articles retained for the review. Click here for file
