# Supplementary material for: Risk factors for non-atopic asthma/wheeze in children and adolescents: a systematic review
Source: Emerg Themes Epidemiol. 2014 Jun 6;11:5. doi: 10.1186/1742-7622-11-5 (PMC4068161; doi:10.1186/1742-7622-11-5)
Supplement: Additional file 2 — Articles retained for the review. [file 1742-7622-11-5-S2.docx]

**Additional file 2 - Articles retained for the review**

| Ref | Author(s) | Country | Study design * | Population | Asthma/wheeze  ascertainment | Atopy definition ** | Endpoint | Confounders | Factors studied | Comparison groups | | | | |
| --- | --- | --- | --- | --- | --- | --- | --- | --- | --- | --- | --- | --- | --- | --- |
|  |  |  |  |  |  |  |  |  |  | Atopic asthma vs non-asthma irrespective of atopy  OR (95% CI) unless otherwise indicated | Atopic asthma vs atopic non-asthma  OR (95% CI) unless otherwise indicated | Atopic asthma vs non-atopic non-asthma  OR (95% CI) unless otherwise indicated | Non-atopic asthma vs non-asthma irrespective of atopy  OR (95% CI) unless otherwise indicated | Non-atopic asthma vs non-atopic non-asthma  OR (95% CI) unless otherwise indicated |
| *13* | Alcântara-Neves NM et al, 2012 | Brazil | Cs | 1,182 4- to 11-year old children | Questionnaire (ISAAC ^[[1]](#footnote-1)^ Phase II) | sIgE | Current wheeze, current wheeze plus symptoms | Yes (sex, age, maternal education, parental asthma) | Burden of infections: light (0 to 3 simultaneous infections) vs heavy (4 to 8) |  | 0.95 (0.60-1.52)^[[2]](#footnote-2)^  1.31 (0.78-2.19)^[[3]](#footnote-3)^ |  |  | 0.87 (0.59-1.29)^5^  0.98 (0.63-1.49)^6^ |
| *14* | Barreto M et al, 2010 | Brazil | Cs | 1,309 4- to 12-year old children | Questionnaire (ISAAC Phase II) and other questions answered by parents | sIgE | Wheeze | Yes (gender, age, motherr’s education, parental asthma, house cleaning, rodents in home, daycare, *T.gondii* infection, *A.lumbricoides* infection, respiratory symptoms, and diarrhea) | Mother’ education (reference: high)  medium  low  Parental asthma  Infrequent household cleaning  Rodents in home  Daycare  *T.gondii* seropositivity  *A.lumbricoides* IgG4  Respiratory symptoms (reference: no)  for 1-7 days  for ≥ 8 days  Diarrhoea (reference: no)  for 1-7 days  for ≥ 8 days |  | 0.94 (0.60-1.46)  1.24 (0.73-2.11)  2.64 (1.48-4.73)  2.27 (0.82-6.29)  0.79 (0.54-1.15)  0.83 (0.49-1.41)  1.51 (0.90-2.54)  1.20 (0.75-1.92)  1.54 (0.92-2.59)  2.27 (0.97-5.38)  1.02 (0.58-1.81)  1.29 (0.70-2.40) |  |  | 1.30 (0.88-1.93)  1.49 (0.93-2.38)  1.91 (1.25-2.92)  2.49 (1.27-4.90)  1.68 (1.21-2.34)  1.52 (1.01-2.29)  1.23 (0.82-1.84)  1.25 (0.79-1.97)  1.54 (1.01-2.36)  4.87 (2.26-9.76)  1.09 (0.67-1.80)  1.59 (0.95-2.66) |
| *17* | Pereira MU et al, 2007 | Brazil | Cs | 1.011 9- to 13-year-old (results reported for 829 non-atopic children) | Questionnaire (ISAAC Phase II) with additional questions | SPT | Current wheeze (wheeze in the past 12 months) and active asthma (wheeze in the past 12 months + asthma ever) | Yes (age and sex) | Maternal asthma  Paternal asthma  Bronchiolitis aged <2 years  Humid household (presently)  Maternal smoking  Born before term  Maternal schooling >8 years  >2 siblings  High load (≥100 eggs g^-1^) *A.lumbricoi des* |  | NR^§§^  NR^§§^  NR^§§^  NR^§§^  NR^§§^  NR^§§^  NR^§§^  NR^§§^  NR^§§^  NR^§§^  NR^§§^  NR^§§^  NR^§§^  NR^§§^  NR^§§^  NR^§§^  NR^§§^  NR^§§^ |  |  | 2.8 (1.6-5.0) ^[[4]](#footnote-4)^  5.4 (2.5-11.7) ^[[5]](#footnote-5)^  3.9 (2.1-7.5) ^28^  3.3 (1.4-7.6) ^29^  4.7 (2.5-8.9) ^28^  14.5 (7.0-30.0) ^29^  1.5 (1.1-2.1) ^28^  2.7 (1.4-5.2) ^29^  1.2 (0.8-1.7) ^28^  1.0 (0.5-1.8) ^29^  1.3 (0.8-2.3) ^28^  0.6 (0.2-1.7) ^29^  0.7 (0.4-1.1) ^28^  0.3 (0.1-0.9) ^29^  1.1 (0.7-1.5) ^28^  0.6 (0.3-1.5) ^29^  2.0 (1.1-3.8) ^28^  3.1 (1.1-6.6) ^29^ |
| *18* | Kurukulaaratchy RJ et al, 2004 | Isle of Wight | C | 1,036 10-year-old children | Parent-completed questionnaire | SPT | Current wheeze (at least on one occasion in the previous 12 months) | Yes (maternal or sibling asthma, social class at birth, chest infections at 1 or 2 yrs, and food allergy at 1 yr, plus (for non-atopic wheeze) maternal urticaria, parental food allergy, cat or dog at birth, exclusive breastfeeding for first 3 months, parental smoking at 4 yrs, eczema at 1 yr and rhinitis at 2 yrs, or (for atopic wheeze) paternal asthma/ eczema/ rhinitis, maternal or sibling rhinitis, family history of urticaria, cat at 1 yr, eczema at 1/2/ 4 yrs, rhinitis at 4 yrs, urticaria at 4 yrs, and gender) | Maternal asthma  Recurrent chest infection at 2 years  Sibling asthma  Eczema at 1 year  Rhinitis at 4 years  Gender (male) |  |  | NR^§§^  NR^§§^  2.10 (1.04-4.23)  2.80 (1.01-7.80)  4.74 (1.61-13.47)  2.73 (1.36-5.48) |  | 4.08 (1.85-9.00)  3.99 (1.78-8.92)  NR^§§^  NR^§§^  NR^§§^  NR^§§^ |
| *19* | Rönmark E et al, 1999 | Sweden | Cs | 2,149 7- to 8-year old children | Questionnaire (ISAAC Phase I with additional questions) | SPT | Ever asthma (current asthma, during the last 12 months, or ex-asthma, present previously) | Yes (family history of asthma, dampness at home, mother smoker, pets at home, breastfeeding, geographical area) | Male gender  Family history of asthma  Dampness at home (past or present)  Mother smoker  Breast-feeding <3 months  Pets at home | 1.62 (1.03-2.54)  2.95 (1.81-4.81)  1.40 (0.81-2.42)  1.17 (0.68-2.01)  1.00 (0.55-1.82)  0.60 (0.36-0.98) |  |  | 1.62 (1.03-2.54)  3.63 (2.33-5.66)  1.78 (1.10-2.89)  1.67 (1.04-2.68)  1.80 (1.11-2.92)  0.75 (0.47-1.21) |  |
| *20* | Sunyer J et al, 2005 | Spain | C | 482 children enrolled at birth (306 had sIgE measured at age 4 years) | Questionnaire | sIgE | Wheeze (“whistling or wheezing from the chest, but not noisy breathing from the nose”) at 4 years of age | Yes (gender, maternal asthma, smoking, parity and education, weeks of gestational age, breastfeeding) | Prenatal exposure to *p-p’* DDE ^[[6]](#footnote-6)^  Maternal asthma  Maternal smoking  Parity (≥ second child)  Gender (male)  Breastfeeding (any) |  | 1.30 (0.91-1.86) # †^[[7]](#footnote-7)^  NR^§§^ # ^[[8]](#footnote-8)^  NR^§§^ #  NR^§§^ #  NR^§§^ #  NR^§§^ #  NR^§§^ # |  |  | 1.37 (1.06-1.79) #† ^39^  2.49 (1.00-6.19) # ^40^  3.45 (1.18-10.10) #  1.03 (0.51-2.10) #  1.54 (0.74-3.24) #  2.84 (1.21-6.68) #  0.34 (0.17-0.69) # |
| *21* | Moncayo AL et al, 2010 | Ecuador | Cs | 3,317 6- to 16-year old children (2,927 healthy, 334 non-atopic wheezers, and 56 atopic wheezers) | Questionnaire (core questions of ISAAC Phase II,plus supplemen tary questions) answered by parents | SPT | Wheeze in the last 12 months | Yes (age and sex) | Gender (male)  Age (reference 6-9 years) 10-12 years  13-16 years  Birth order ≥ 4 vs <4  *T.trichiura* infection  ≤ median (490 eggs/g) vs no  > median vs no  Watching TV > 3hrs/day vs no  Frequency of exercise once a week or less vs daily  Daycare <1 year old vs no  ≥1 year old vs no  Contact with animals on farms  Maternal allergic disease  Maternal education level (illiterate or incomplete primary vs complete second ary or higher)  Mother smoking presently  Mother smoking in pregnancy  Excreta disposal (open field)  Family monthly income (≤150 US$)  Cat in house presently  Cat in house ever |  |  | 2.73 (1.44-5.16)  1.34 (0.70-2.57)  0.95 (0.40-2.28)  0.61 (0.29-1.28)  0.49 (0.24-1.01)  0.24 (0.09-0.63)  0.86 (0.32-2.30)  0.95 (0.36-2.48)  0.66 (0.20-2.19)  1.04 (0.52-2.09)  1.04 (0.60-1.81)  1.64 (0.78-3.42)  0.53 (0.11-2.615  1.10 (0.39-3.12)  2.18 (0.63-7.48)  1.16 (0.57-2.38)  1.29 (0.71-2.36)  1.50 (0.86-2.63)  1.45 (0.85-2.46) |  | 0.91 (0.71-1.16)  0.56 (0.42-0.74)  0.39 (0.25-0.62)  0.71 (0.57-0.88)  1.00 (0.74-1.35)  1.00 (0.70-1.43)  1.51 (1.06-2.16)  1.32 (0.81-2.14)  0.81 (0.55-1.19)  1.08 (0.85-1.38)  1.01 (0.74-1.39)  3.24 (2.42-4.32)  0.80 (0.49-1.30)  1.46 (0.83-2.56)  0.94 (0.53-1.67)  1.31 (0.94-1.83)  0.78 (0.60-1.01)  0.76 (0.56-1.02)  1.25 (0.94-1.66) |
| *22* | Janson C et al, 2007 | Sweden | Cs | 195 13- to 14-year-old children (99 non asthmatic; 48 atopic asthmatic; 48 non-atopic asthmatic) | Parents’ report of current asthma, or affirmative answers to specific questions of self-completed questionnaire (ISAAC Phase II plus additional questions), or high exhaled NO; followed by clinical assessment | SPT | Current asthma | Yes (sex, BMI, parental asthma, and type of housing) | Gender (female)  BMI (per 5 units increase)  Parental asthma  Early life infections: Croup  Otitis (≥5 episodes/year)  Cat or dog when infant  Window pane condensation (current)  Dirty school (currently) | 0.82 (0.45-1.49)  1.62 (1.06-2.48)  2.16 (1.12-4.18)  1.46 (0.72-2.98)  0.78 (0.33-1.79)  1.48 (0.78-2.78)  1.07 (0.36-3.12)  1.40 (0.70-2.80) |  |  | 1.43 (0.78-2.63)  1.55 (1.02-2.36)  2.35 (1.23-4.52)  2.80 (1.44-5.43)  1.99 (1.02-3.88)  2.17 (1.16-4.04)  2.45 (1.11-5.40)  2.50 (1.28-4.89) |  |
| *23* | Civelek, E et al, 2011 | Turkey | Cs | 5,461 children, mean age 10.8 (SEM not reported) years | Questionnaire (including ISAAC Phase II questions), self-completed by parents | SPT | Current wheeze ( “Has your child had wheezing or whistling in the chest in the past 12 months?”) | Yes (the same as reported as risk factors, after backward elimination from the full set of variables) | Gender (male)  Prematurity  History of parental asthma/rhinitis  Dampness/mold in 1^st^ year of life  over the past year  Crowding (one-room house)  Maternal smoking in pregnancy  Secondhand smoke over the past year  Current rhinoconjunctivitis  Atopic dermatitis | NR^§§^  2.08 (1.05–4.13)  1.87 (1.13–3.11)  NR^§§^  NR^§§^  NR^§§^  1.72 (1.05–2.83)  NR^§§^  2.95 (2.02–4.32)  NR^§§^ |  |  | 1.26 (1.00-1.59)  NR^§§^  1.74 (1.30–2.32)  1.48 (1.06–2.08)  2.44 (1.73–3.44)  2.24 (1.48–3.39)  1.43 (1.08–1.89)  1.63 (1.28–2.09)  2.49 (2.10–2.95)  2.16 (1.63–2.88) |  |
| *24* | Garcia-Marcos L et al, 2005 | Spain | Cs | 2,970 9- to 12-year-old children | Questionnaire (ISAAC Phase II) | SPT | Current wheeze | Yes (gender, maternal or paternal asthma, mother smoking 1^st^ year, mould stains currently) | Gender (male)  Paternal or maternal asthma  Mother smoking (1^st^ year)  Mould stains currently  Cat ownership currently  Dog ownership (1^st^ year) |  | 1.56 (1.11-2.19)  2.10 (1.39-3.18)  1.12 (0.80-1.56)  1.78 (0.78-4.10)  1.58 (0.94-2.67)  1.22 (0.75-1.96) |  |  | 1.15 (0.78-1.71)  1.39 (0.80-2.42)  1.74 (1.17-2.58)  2.66 (1.13-6.25)  0.66 (0.31-1.41)  1,29 (0.75-2.23) |
| *25* | Weinmayr G et al, 2013 | 20 countries of ISAAC Phase II | Cs | 46,051 8-12-year old children, of whom 6,633 atopic and 19,786 non-atopic | Questionnaire (ISAAC Phase I) and additional questions on dampness and mould | SPT | Wheeze in the past year | Yes (parental allergic disease) | Damp/mould currently present in the household |  | 1.69 (1.42-2.02) |  |  | 1.54 (1.33-1.77) |
| *26* | Pekkanen J et al, 2007 | Finland | Cc | 306 1- to 8-year old children (62 atopic cases, with 124 controls, and 40 non-atopic cases, with 80 controls) | Physician-diagnosed asthma | sIgE | Asthma (earlier attack(s) of asthma must not have occurred > 1 yr previously) | Yes (parental asthma, father’s education, number of siblings, pets indoors currently, day-care) | Moisture damage in main living area  Visible mould in main living area  Moisture damage in child’s bedroom | 2.84 (1.15-5.05)  4.74 (0.94-24.10)  2.10 (0.73-6.01) |  |  | 2.11 (0.68-6.53)  1.08 (0.32-3.64)  1.89 (0.57-6.25) |  |
| *27* | Venn AJ et al, 2003 | UK | Cc | 416 9- to 11-year-old children (193 wheezers and 223 non-wheezers) | Parents’ interview | SPT | Wheeze in the past year (both at 6-8 years of age and at 9-11 years of age) | Yes (age, sex, socioeconomic status) | Damp (in the living room) ^[[9]](#footnote-9)^ |  | 1.51 (0.95-2.40) |  |  | 1.02 (0.65-1.61) |
| *28* | Oddy WH et al, 2002 | Australia | Cs | 221 6-year-old children | Parent-completed questionnaire | SPT | Current asthma at 6 years of age (doctor-diagnosed asthma-ever with  wheeze or cough without a cold in the last year, and currently taking either preventer or reliever asthma medication) | Yes (gestational age <37 weeks, maternal smoking in pregnancy, sex, duration of exclusive breast feeding) | Wheezing LRI in 1^st^ year of life (≥ 2 vs none) | 9.00 (5.25-15.42) |  |  | 4.10 (2.48-6.77) |  |
| *29* | Normann E et al, 2006 | Sweden | Cs | 1,581 (827 boys, 754 girls) 4- to 5.4-year-old children | Parent-completed questionnaire | sIgE | Wheeze at 4 years of age (at least one exacerbation of wheezing during the last year) | Not reported | anti-*Chlamydia pneumoniae* IgG, in girls (assessed at age 4 years) |  | 1.13 (0.34-3.70) ^††^ |  |  | 2.39 (1.25-4.57) ^††^ |
| *30* | Kusel MMH et al, 2007 | Australia | C | 198 5-year-old children | Wheeze reported by parents | SPT | Current wheeze (wheeze in in 12 months before the 5-year visit) | Reported associations are not adjusted; however, they reportedly remain after adjusting for gender, parental atopic disease, pet ownersh ip, older siblings, environmental smoke exposure, and daycare atten dance | Any wheezy LRI in 1^st^ year  Any febrile LRI  Any wheezy LRI with rhinovirus |  | 3.4 (1.2-9.7) ^[[10]](#footnote-10)^  0.5 (0.1-3.5) ^[[11]](#footnote-11)^  4.2 (1.5-11.8) ^26^  1.3 (0.2-9.9) ^27^  3.2 (1.1-9.5) ^26^   - 1. (0.3-18.5) ^27^ |  |  | 1.4 (0.4-5.1)  1.0 (0.2-3.8)  1.6 (0.3-8.7) |
| *31* | Alcântara-Neves NM et al, 2010 | Brazil | Cs | 148 non-atopic and 135 atopic children, less than 5 years old | Questionnaire (ISAAC Phase II) and interview with parents | sIgE | Wheeze (4+ episodes associated with cough, breath shortness and asthma medication in the past 12 months) | Yes (gender, age, *T.trichiura* eggs, *A.lumbricoides* eggs, and days with diarrhea) | *T.trichiura* active infection  *A.lumbricoides* active infection  Anti-*Ascaris* IgE  Diarrhoea (6+ days vs none) |  | 3.07 (1.00-9.43)  0.44 (0.15-1.32)  2.01 (1.00-4.50)  1.62 (0.53-4.89) |  |  | 2.45 (0.70-8.53)  0.76 (0.27-2.11)  3.07 (1.13-8.35)  1.79 (0.55-5.78) |
| *32* | von Mutius E et al, 1999 | Germany | Cs | 11,094 5- to7-year-old and 9- to11-year-old children (speci fic numbers of asthmatics / non-asthmatics and atopics / non-atopics not clearly presented) | Questionnaire (ISAAC Phase I) | sIgE | Asthma (parents reported that asthma had been diagnosed at least once or that a doctor had diagnosed asthmatic, spastic or obstructive bronchitis more than once) | Yes (family history of atopy, number of siblings, parental educa tion, school grade, study area) | No of fever episodes in the 1^st^ year  1-2 vs none  3-4 vs none  ≥5 vs none  No of antibiotic courses in the 1^st^ 3 years  1-2 vs none  3-5 vs none  ≥6 vs none | 1.03 (0.69-1.55)  4.33 (0,83-2.14)  2.31 (1.43-3.74)  1.10 (0.70-1.73)  1.69 (1.06-2.70)  4.38 (2.73-7.01) |  |  | 2.18 (1.14-4.16)  4.50 (2.32-8.88)  11.48 (5.95-22.12)  2.39 (1.13-5.08)  6.82 (3.32-14.00)  24.29 (11.86-49.76) |  |
| *33* | Calvani M et al | Italy | Cc | 338 asthma cases (mean age 6.9±3.1 years) and 467 controls (mean age 6.7±3.0 years) | Parents’ interview | SPT | Asthma (history of 3+ episodes of wheezing requiring ß2 agonists or diagnosis of asthma by a doctor) | Yes (gender, age, maternal asthma, and maternal smoking in pregnan cy, plus rhinitis (for ‘flu’, fever and isoxsuprine in non-atopics) or atopic dermatitis (for fever in atop ics and threatened abortion in non-atopics). No reported confounders for antibiotic courses | ‘Flu’ episodes in pregnancy  Fever episodes in pregnancy  Antibiotic courses in pregnancy  Threatened abortion/premature labour  Isoxsuprine use in pregnancy | NR^§§^  2.24 (1.17-4.30)  1.71 (0.90-3.24) ^††^  NR^§§^  NR^§§^ |  |  | 2.28 (1.15-4.52)  2.18 (0.98-4.86)  1.51 (0.68-3.36) ^††^  1.66 (0.96-2.86)  1.87 (1.12-3.12) |  |
| *34* | Fuchs O et al, 2012 | Germany, Austria, Switzerland | Cs | 8,023 6- to 12-year-old children (2,977 farm children, 2,660 exposed (i.e., to stables, barns or unprocessed cow’s milk) non-farm children, 2,386 unexposed children) | Questionnaire | sIgE | Transient wheeze, , current wheeze (persistent wheeze + late-onset wheeze) | Yes (study center, sex, age, family history of allergic disease) | Living in a farm vs not living in a farm and being unexposed to stables, barns or unprocessed cow’s milk |  | NR^§§^  NR^§§^ |  |  | 0.45 (0.32-0.63)^[[12]](#footnote-12)^  0.38 (0.24-0.58)^[[13]](#footnote-13)^ |
| *35* | Ege MJ et al, 2007 | Austria, Germ any, The Nethelands, Sweden, Switzerland | Cs | 2,086 5- to 13-year-old children, part of the 2,823 farm children + 5,440 reference children in the overall study, who were blood tested | Questionnaire (ISAAC Phase II, and additional questions) | sIgE | Asma ever (reported doctor-diagno sed asthma once, or obstructive bronchitis >1 in lifetime) | Yes (type of farm, animal species kept, animal feed, number of older siblings, sex, study center, family history of asthma) | Use of silage |  |  |  | 0.55 (0.31-0,98) |  |
| *36* | Braun-Fahrländer C et al, 2002 | Austria, Germany. Switzerland | Cs | 812 6- to 13- year-old children (mean (SD) age 9.5 (1.2) years), 319 from farming families and 493 from non-farming families | Parent-completed questionnaire (ISAAC Phase I) and additional questions on farming | sIgE | Asthma ( parents reported a doctor-diagnosed asthma or children had recurrent asthmatic airway obstructi on or spastic bronchitis); wheeze (parents reported that children had wheezing /whistling in the chest the previous 12 months) | Yes (age, sex, study area, family history of asthma or hay fever, educational level of the parents, number of older siblings, and, for endotoxin exposure, exposure to farming in the 1^st^ year of life) | Exposure to farming in the 1^st^ year of life  Endotoxin load (units/m^2^ of mattress surface)  Prevalence (% (95% CI)) of outcome according to farming status  Farming  Nonfarming | 0.42 (0.18-0.96) ^[[14]](#footnote-14)^  0.59 (0.28-1.23) ^[[15]](#footnote-15)^  0.52 (0.30-0.90) ^[[16]](#footnote-16)^  0.66 (0.41-1.07) ^[[17]](#footnote-17)^  3.1 (1.2-5.0) ^41^  4.7 (2.4-7.0) ^42^  5.9 (3.8-8.0) ^41^  5.9 (3.8-8.0) ^42^ |  |  | 0.48 (0.16-1.41) ^41^  0.43 (0.19-0.97) ^42^  1.22 (0.60-2.46) ^43^  1.23 (0.73-2.06) ^44^  1.6 (0.2-2.9) ^41^  1.6 (0.2-2.9) ^42^  2.6 (1.2-5.0) ^41^  6.1 (4.0-8.2) ^42^ |  |
| *38* | Kelley CF et al, 2005 | USA | Cs | 5,244 6- to 16-year-old children (representative sample of ≈39.6 million US children) | Questionnaire | SPT | Current asthma (“Has a doctor ever told you that your child has asthma?”, followed by “Does the child still have asthma?”) | Yes (age, gender, race / ethnicity, Poverty/Income Ratio, education, BMI, cotinine level, prenatal maternal smoking, child care attendance) | PIR (Poverty/Income ratio) ^[[18]](#footnote-18)^  Schooling of adult respondent (years)  BMI percentile  Gender (female)  Prenatal maternal smoking  Nonsmokers cotinine level, ng/mL  Child care attendance | 2.0 vs 2.2 ^[[19]](#footnote-19)^ (p< 0.5)  12.4 vs 12.0 ^37^ (p< 0.5)  62.7 vs 57.7 ^37^ (NS) ^§^  44.1 vs 49.0 ^[[20]](#footnote-20)^ (NS) ^§^  10.5 vs 13.4 ^38^ (NS) ^§^  0.8 vs 0.7 ^37^ (NS) ^§^  39.9 vs 34.9 ^38^ (NS) ^§^ |  |  | 2.4 vs 2.2 ^37^ (NS)^§^  12.2 vs 12.0 ^37^ (p< 0.5)  68.5 vs 57.7 ^37^ (p< 0.5)  54.4 vs 49.0 ^38^ (NS) ^§^  14.4 vs 13.4 ^38^ (NS) ^§^  0.6 vs 0.7 ^37^ (NS) ^§^  37.6 vs 34.9 ^38^ (NS) ^§^ |  |
| *39* | Visness CM et al, 2010 | USA | Cs | 3,387 2- to 19-year olds | Interview to participants, or proxies if under age 16. | sIgE | Current asthma (doctor or other health professional ever said the child had asthma + the child still has asthma), wheeze (report of wheeze in the past year) | Yes (age, survey round, race/ethnicity, sex, poverty income ratio, household smoking, level of physical activity) | Overweight^[[21]](#footnote-21)^  Obesity^[[22]](#footnote-22)^  Increase of Body Mass Index (BMI) ^[[23]](#footnote-23)^  C-reactive Protein (CRP) ^[[24]](#footnote-24)^ | 1.05 (0.76-1.45) ^[[25]](#footnote-25)^  0.97 (0.69-1.38) ^[[26]](#footnote-26)^  1.34 (0.70-2.57) ^17^  0.85 (0.54-1.36) ^18^  1.13 (0.91-1.41) ^17^  0.97 (0.65-1.44) ^18^† |  |  | 1.29 (0.65-2.53) ^17^  1.52 (0.77-2.98) ^18^  2.46 (1.21-5.02) ^17^  2.20 (1.15-4.22) ^18^  1.52 (1.14-2.04) ^17^  1.45 (1.61-1.81) ^18^† |  |
| *40* | Lødrup-Carlsen KC et al, 2012 | Sweden, The Netherlands | C | 3,719 and 2,620 6- to 12-year-old children (in Sweden and The Netherlands, respectively) enrolled at birth, for whom data on pet ownership at age 0-2 years were reported; number of children with non-atopic asthma not reported | Questionnaire | sIgE | Current asthma, as satisfying at least 2 out of 3 parent-reported conditions: (i) doctor-diagnosed asthma ever; (ii) asthma symptoms /wheezing (last 12 months) according to the ISAAC core questions; (iii) using asthma medication (last 12 months) | Yes (family history of asthma and/or allergic rhinitis; family history of pet allergy; maternal smoking during pregnancy; postnatal maternal smoking from after birth to last follow-up between 6 to 10 years of age; educational level of parents at birth of child; one or more older siblings; home/apartment with convenient ground access; crowding at home; gender; breast feeding duration; doctor’s diagnosed eczema any time between birth and 2 years | Both dog(s) and cat(s) ownership at age 0-2 years |  |  | NR^§§^  NR^§§^ |  | 3.66 (1.50-8.93) |
| *41* | Ponsonby AL et al, 2002 | Australia | Cs | 722 8- to 10-year-old children | Questionnaire (ISAAC Phase I with additional questions) | SPT | Asthma ever, recent (past year) wheeze, persistent (>12 episodes in past year) wheeze | Yes (N of siblings, breastfeeding, maternal smoking in same room as child, home gas heater) | Use of synthetic quilt  Increasing synthetic upper bedding ^[[27]](#footnote-27)^ |  |  | 1.67 (1.05-2.65) ^[[28]](#footnote-28)^  1.63 (1.03-2.59) ^[[29]](#footnote-29)^  3.47 (1.33-9.07) ^[[30]](#footnote-30)^  1.25 (1.01-1.54) ^47^  1.21 (0.98-1.49) ^48^  1.69 (1.08-2.64) ^49^ |  | 1.15 (0.72-1.83) ^47^  1.27 (0.71-2.27) ^48^  0.45 (0.04-4.72) ^49^  1.04 (0.84-1.29) ^47^  1.05 (0.80-1.38) ^48^  0.77 (0.26-2.21) ^49^ |
| *42* | Kozyrskyj AL et al, 2009 | Australia | C | 1,999 6-year old children (1,365 in non-atopic asthma analysis), assessed again at age 14 (1,693 children, 1,390 n non-atopic asthma analysis) | Parent-completed questionnaire | SPT | Current reported asthma (dr-diagno sed asthma ever + asthma symptoms in the past 12 months + current use of asthma medications) | Yes (gender, maternal asthma, family stress, prematurity, dog ownership, wheeze during sleep and co-sleeping, at 6 years; matern al asthma, family stress, cat owner ship, single-parent status, wheeze during sleep and co-sleeping, at 14 years) | Frequent nocturnal awakenings persi sting over the first 3 years of life |  |  |  | 1.87 (1.08-3.25) ^[[31]](#footnote-31)^  2.18 (1.15-4.13) ^[[32]](#footnote-32)^ |  |
| *43* | Gruzieva O et al, 2013 | Sweden | C | Birth cohort assessed for asthma at 4 years (2,008 children) and 8 years of age (1,692 children) | Questionnaire | sIgE | Asthma, defined as at least four episodes of wheeze in the last 12 months, or at least one episode in combination with prescription of inhaled corticosteroids | Yes (municipality, socioeconomic status, heredity, and the year the house was built) | Exposure to PM_10_ and NO_x_ during the 1^st^ year of life | 1.4 (0.3-6.8) ^[[33]](#footnote-33)^  1.5 (0.4-5.1) ^[[34]](#footnote-34)^  1.1 (0.3-3.8) ^[[35]](#footnote-35)^  0.8 (0.2-2.4) ^[[36]](#footnote-36)^ |  |  | 1.6 (0.5-5.3) ^1^  2.4 (1.0-5.6) ^2^  3.8 (0.9-16.2) ^3^  2.6 (0.9-8.1) ^4^ |  |
| *44* | Palmieri M et al, 1990 | Italy | Cc | 302 1- to 12-year-old non-atopic asthmatic children, and 433 matched healthy controls (elements of matching not reported, except the geographical region of origin) | GP’s diagnosis | SPT | Asthma | none | Parental heavy smokers (20+ cigarettes /day) |  |  |  | 62% vs 42% (p=0.028) ^[[37]](#footnote-37)†^  67% vs 40% (p=0.024) ^[[38]](#footnote-38)†^ |  |
| *45* | Yoo S et al, 2009 | South Korea | Cs | 724 15- to 16-year-old children (251 non-atopic no smokers, 25 non-atopic smokers, 401 atopic no smokers, 47 atopic smokers) | Self-completed questionnaire (ISAAC, written and video) and additional questions on active and second-hand smoke | SPT | Wheeze ever, wheeze in the last 12 months, exercise-induced wheeze in the last 12 months, among others | Yes (sex, parents’ smoking, contact with peers who smoked) | Active current smoking (smoking >1 day in the 30 previous days) |  |  | 1.1 (0.4-2.9) ^[[39]](#footnote-39)a^  1.4 (0.5-4.0) ^23b^  1.4 (0.3-5.6) ^[[40]](#footnote-40)a^  1.4 (0.3-8.0) ^24b^  1.2 (0.3-4.7) ^[[41]](#footnote-41)a^  5.3 (1.7-16.7) ^25b^ |  | 2.6 (0.9-7.6) ^23a^  3.1 (1.1-9.2) ^23b^  6.5 (2.0-21.0) ^24a^  8.9 (2.2-36.2) ^24b^  10.3 (3.4-31.0) ^25a^  17.1 (4.9-60.1) ^25b^ |
| *46* | Genuneit J et al, 2006 | Germany | C | 2,092 (767 atopic, 1,325 non-atopic) 16- to 18-year-old adolescents | Questionnaires (ISAAC Phase II at baseline, and ECRHS^[[42]](#footnote-42)^ at follow-up) | SPT/sIgE | Wheeze, wheeze without a cold, diagnosed asthma | Yes (sex, age, study center, duration of exposure to ETS) | Active smoking in adolescence  Low baseline α_1_-antitrypsin level |  | 1.63 (1.20-2.22) # ^[[43]](#footnote-43)^  2.07 (1.35-3.18) # ^[[44]](#footnote-44)^  2.16 (1.15-4.06) # ^[[45]](#footnote-45)^  1.33 (1.02-1.73) # ^32,^^[[46]](#footnote-46)^  1.63 (1.20-2.22) # ^33,35^  NR^§§ 13,34,35^ |  |  | 3.25 (2.22-4.76) # ^32^  3.92 (2.01-7.65) # ^33^  2.16 (0.79-5.93) # ^34^  NR^§§^ # ^32,35^  NR^§§^ # ^33,35^  1.72 (0.96-3.06) # ^34,35^ |
| *47* | de Meer G et al, 2010 | The Nether lands | Cs | 1,547 8- to 13-year old children (1,239 non-atopic non-wheezers, 143 non-atopic wheezers, and 165 atopic wheezers) | Questionnaire (ISAAC) and other questions answered by parents | SPT/sIgE | Current wheeze | Yes (age and sex) | High parental education |  |  | 0.89 (0.60-1.31) |  | 0.65 (0.43-0.99) |
| *48* | Burr et al, 1993 | UK | C | 453 7-year-old children 274 non-atopics) | Interview | SPT | Wheeze (‘a whistling sound, wheth er high or low pitched, and however faint’) during the past year | Wheeze at 7 years of age | Breast fed (ever vs never) |  | NR^§§^ |  |  | 0.52 (0.27-0.98) |
| *49* | Nagel G et al, 2009 | 20 countries | Cs | 31,759 8- to 12-year old children, with SPT perform ed | Parent-completed questionnaire (ISAAC Phase I) and additional questions on breastfeeding | SPT | Wheeze during the previous year | Yes (adjusted for sex, age, maternal atopic disease, ETS and present bedroom sharing) | Breastfeeding (any) |  | 0.85 (0.54-1.34) ^[[47]](#footnote-47)^  0.85 (0.67-1.08) ^[[48]](#footnote-48)^ |  |  | 0.69 (0.53-0.90) ^21^  0.87 (0.72-1.06) ^22^ |
| *50* | Nwaru BI et al, 2013 | Finland | Cc | 3,142 5-year-old children | Questionnaire | sIgE | Asthma, as doctor-diagnosed asthma plus either any wheezing symptom or use of asthma medication during the preceding 12 months | Yes (sex, parental asthma, parental rhinitis, No of siblings, hospital of birth, maternal smoking in pregnancy, and other variables based on statistical significance) | Breastfeeding (reference:>9 months)  for <5.0 months  for 5.0-9.5 months | NR^§§^  NR^§§^ |  |  | 2.95 (1.31-6.66)  3.60 (1.67-7.76) |  |
| *51* | Remes ST et al, 2008 | Finland | C | 4,647 (489 asthmatic) 16-year-old children | Self-completed questionnaire | SPT | Self-report of doctor-diagnosed asthma ever | No (none of potential confounders changed the estimates more than 10%) | Asthma |  | 2.40 (1.33-4.32) |  |  | 0.99 (0.38-2.54) |
| *52* | Pike KC et al, 2010 | UK | C | 1,164 3 year-old children (555 non-atopic wheezers, 127 atopic wheezers, 67 atopic non-wheezers, 415 non-atopic non-wheezers) | Interview to mothers for evidence of early childhood wheeze | SPT | Wheeze at age 3 years (‘any episodes of chestiness associated with wheezing or whistling in his/her chest since they were last seen’) | Yes (adjusted for gender, maternal age, smoking in pregnancy, maternal asthma, maternal rhinitis, paternal asthma, maternal education and birth order) | Birthweight^[[49]](#footnote-49)^  11-19-gestation week head circumfe rence growth ^12^  11-19-wk abdomen circumfer. growth ^12^  19-34-wk head circumference growth ^12^  19-34-wk abdomen circumfer. growth ^12^  0-6 months weight growth ^12^  0-6 mo subscapular skinfold growth ^12^  6-12 months weight growth ^12^  6-12 mo subscapular skinfold growth ^12^ |  |  | 1.02 (0.87-1.19) #  0.79 (0.54-1.15) #  1.32 (0.94-1.85) #  0.88 (0.69-1.12) #  0.80 (0.65-1.00) #  1.22 (0.92-1.03) #  1.27 (1.03-1.03) #  1.19 (0.94-1.31) #  1.20 (1.03-1.39) # |  | 0.97 (0.92-1.03) #  0.90 (0.81-1.00) #  0.97 (0.87-1.09) #  0.94 (0.88-1.01) #  1.05 (0.98-1.13) #  1.04 (0.99-1.10) #  1.06 (1.00-1.11) #  1.08 (1.00-1.15) #  1.02 (0.96-1.09) # |
| *53* | Pike KC et al, 2011 | UK | C | 113 3-year-old children, 78 of whom with FEV_0.4_ measured | Parent-reported wheeze | SPT | Wheeze at age 3 years | Yes (age) | FEV_0.4_ ^[[50]](#footnote-50)^  at age 5–14 weeks |  |  | 148.9 (110.1-201.3) ml vs 139.0 (132.2-146.2) ml, % difference 7.1 (-9.6 to 26.9) |  | 123.7 (114.6-133.5) ml vs 139.0 (132.2-146.2) ml, % difference -11.0 (-19.2 to -2.0) |
| *54* | Knudsen TB et al, 2007 | Denmark | Cs | 1,007 7- to 17-year-old children | Interview (questions adapted from the American Thoracic Society) | SPT | Asthma | Several potential confounders listed in Methods, but their use for adjust ting reported ORs is not explicitly stated | Being born in autumn (vs the rest of the year) | 2.41 (1.25-4.64) |  |  | 2.35 (1.14-4.83) |  |
| *55* | BahreinianS et al, 2011 | Canada | Cs | 431 11- to 14- year old children (136 with asthma and 295 without asthma) | Parent-reported (by mail-out survey) and doctor-confirmed asthma | SPT | Asthma | Yes (ethnicity, increase of waist circumference, gender) | Depression, overall  girls  boys |  |  | 1.35 (0.75-2.43)  1.10 (0.45-2.71)  1.45 (0.65-3.22) |  | 2.47 (1.12-5.44)  2.84 (1.00-8.10)  1.95 (0.58-6.51) |
| *56* | Kohlboeck G et al, 2013 | Germany | Cr | 2,814 participants (1,374 girls, 1,440 boys) assessed at age 10 years for asthma ever | Question to parents (‘Has your child ever had a diagnosis of asthma according to a physician?’) | sIgE | Asthma ever | Yes (gender, study center, parental educational level, household income, body mass index (BMI), other allergic diseases, and pubertal status) | Abnormal emotional problems  overall  girls  boys | 1.35 (0.81-2.26)  1.08 (0.43-2.74)  1.56 (0.83-2.93) |  |  | 2.90 (1.46-5.73)  3.18 (1.23-8.23)  2.37 (0.86-6.55) |  |
| *57* | Cookson H et al, 2009 | UK | C | 5,810 7 ½ year-old children (5,075 non-asthmatics, 271 atopic asthmatics, 228 non-atopic asthmatics, 236 asthmatics without assessm ent of atopic status) | Questionnaire sent to mothers at 91 months after birth | SPT | Current asthma at age 7 ½ years (doctor’s diagnosis of asthma ever and either reported symptoms of wheeze or treatment for asthma in the previous 12 months) | Yes (sex, preterm delivery, multiple birth, number of siblings, maternal age, maternal education, maternal history of asthma and allergy, prenatal tobacco smoke exposure, and problems during pregnancy (diabetes, hypertension, steroid intake)) | Maternal anxiety  at 18 weeks of pregnancy  2^nd^ quartile (ref: 1^st^ quartile)  3^rd^ quartile (ref: 1^st^ quartile)  4^th^ quartile (ref: 1^st^ quartile)  at 32 weeks of pregnancy  2^nd^ quartile (ref: 1^st^ quartile)  3^rd^ quartile (ref: 1^st^ quartile)  4^th^ quartile (ref: 1^st^ quartile) | 1.27 (0.91-1.77)  1.11 (0.79-1.56)  1.20 (0.83-1.74)  1.08 (0.76-1.54)  1.27 (0.90-1.77)  1.43 (1.00-2.05) |  |  | 0.92 (0.91-1.77)  1.17 (0.91-1.77)  1.78 (0.91-1.77)  1.57 (1.06-2.33)  1.72 (1.17-2.51)  1.80 (1.20-2.70) |  |
| *58* | Marques dos Santos D et al, 2012 | Brazil | Cs | 1,013 4- to 12-year old children | Questionnaire (ISAAC Phase II) answered by parents | sIgE | Asthma (wheezing in the last 12 months associated with at least one of the following: medical diagnosis of asthma, difficult talking due to wheezing, waking in the night at least once a week, wheezing after physical exercise) | Yes (child’s age and history of pneumonia, mother’s education and history of asthma, exposure to mould) | Suspected mother’s CMD ^[[51]](#footnote-51)^  Social support:  affective  material  emotional  informational  social interaction |  |  | 1.74 (1.12-2.71)  0.85 (0.52-1.37)  0.76 (0.48-1.20)  0.82 (0.50-1.35)  0.96 (0.60-1.55)  1.02 (0.64-1.63) |  | 1.73 (1.17-2.55)  0.67 (0.45-1.01)  0.63 (0.42-0.95)  0.88 (0.57-1.34)  0.60 (0.40-0.90)  0.69 (0.46-1.03) |

* Cc case-control, Cs cross-sectional, C cohort

** SPT skin prick test

# RR (95% CI)

§ non-significant

§§ not reported

† not adjusted

†† not reported whether adjusted or not

1. International Study of Asthma and Allergies in Childhood [↑](#footnote-ref-1)
2. current wheeze [↑](#footnote-ref-2)
3. current wheeze with symptoms [↑](#footnote-ref-3)
4. wheeze [↑](#footnote-ref-4)
5. active asthma [↑](#footnote-ref-5)
6. dichlorodiphenyldichloroetylene [↑](#footnote-ref-6)
7. for each doubling of *p-p’* DDE [↑](#footnote-ref-7)
8. for concentration of *p-p’* DDE in cord serum of >1.90 ng/ml compared to <0.57 [↑](#footnote-ref-8)
9. OR per increasing category of damp, from very low to high [↑](#footnote-ref-9)
10. atopic by age of 2 years [↑](#footnote-ref-10)
11. atopic after age of 2 years [↑](#footnote-ref-11)
12. current wheeze (persistent wheeze (onset before age 3 years and wheeze at school age) or late-onset wheeze (≥ age 3 years)) [↑](#footnote-ref-12)
13. persistent wheeze [↑](#footnote-ref-13)
14. asthma [↑](#footnote-ref-14)
15. wheeze [↑](#footnote-ref-15)
16. OR (95% CI) for the occurrence of asthma with increase of endotoxine load from the lowest quartile to the highest quartile [↑](#footnote-ref-16)
17. OR (95% CI) for the occurrence of wheeze with increase of endotoxine load from the lowest quartile to the highest quartile [↑](#footnote-ref-17)
18. ratio of income to family's poverty threshold, calculated by dividing family income by the poverty guidelines, specific to family size, year and state (US Census Bureau) [↑](#footnote-ref-18)
19. arithmetic mean [↑](#footnote-ref-19)
20. percentage [↑](#footnote-ref-20)
21. overweight (≥85% to <95% percentile of BMI-for-age) [↑](#footnote-ref-21)
22. obesity (≥95% percentile of BMI-for-age) [↑](#footnote-ref-22)
23. SD increase in the BMI *z*-score [↑](#footnote-ref-23)
24. 1-log increase of CRP [↑](#footnote-ref-24)
25. current asthma [↑](#footnote-ref-25)
26. wheeze in the past year [↑](#footnote-ref-26)
27. increase in one category across four categories (no synthetic pillow nor quilt, synthetic pillow only, synthetic quilt only, both synthetic pillow and quilt) [↑](#footnote-ref-27)
28. asthma ever [↑](#footnote-ref-28)
29. recent wheeze (in past year) [↑](#footnote-ref-29)
30. persistent wheeze (>12 episodes in past year) [↑](#footnote-ref-30)
31. at age 6 years [↑](#footnote-ref-31)
32. at age 14 years [↑](#footnote-ref-32)
33. asthma at age 4 years for a change in exposure corresponding to 5^th^ to 95^th^ percentile difference in the estimated outdoor level of PM_10_ during the 1^st^ year of life (7.2 μg/m^3^) [↑](#footnote-ref-33)
34. asthma at age 4 years for a change in exposure corresponding to 5^th^ to 95^th^ percentile difference in the estimated outdoor level of NO_x_ during the 1^st^ year of life (46.8 μg/m^3^) [↑](#footnote-ref-34)
35. asthma at age 8 years for a change in exposure corresponding to 5^th^ to 95^th^ percentile difference in the estimated outdoor level of PM_10_ during the 1^st^ year of life (7.2 μg/m^3^) [↑](#footnote-ref-35)
36. asthma at age 8 years for a change in exposure corresponding to 5^th^ to 95^th^ percentile difference in the estimated outdoor level of NO_x_ during the 1^st^ year of life (46.8 μg/m^3^) [↑](#footnote-ref-36)
37. among 1-6 years old children (chi2= 7.10, 2 DF) [↑](#footnote-ref-37)
38. among 3-6 years old children (chi2= 7.40, 2 DF) [↑](#footnote-ref-38)
39. wheeze ever (^a^ written questionnaire, ^b^ video questionnaire) [↑](#footnote-ref-39)
40. wheeze in the last 12 months (^a^ written questionnaire, ^b^ video questionnaire) [↑](#footnote-ref-40)
41. exercise-induced wheeze in the last 12 months (^a^ written questionnaire, ^b^ video questionnaire) [↑](#footnote-ref-41)
42. European Community Respiratory Health Survey [↑](#footnote-ref-42)
43. wheeze [↑](#footnote-ref-43)
44. wheeze without a cold [↑](#footnote-ref-44)
45. diagnosed asthma [↑](#footnote-ref-45)
46. in smokers [↑](#footnote-ref-46)
47. non-affluent countries [↑](#footnote-ref-47)
48. affluent countries [↑](#footnote-ref-48)
49. SD increase [↑](#footnote-ref-49)
50. Forced Expiratory Volume in 0.4 sec; geometric mean (95% CI) in wheezers vs non-wheezers [↑](#footnote-ref-50)
51. Common Mental Disorder [↑](#footnote-ref-51)
